# Experimental Study on Damage Detection in ECC-Concrete Composite Beams Using Piezoelectric Transducers

**DOI:** 10.3390/s19122799

**Published:** 2019-06-22

**Authors:** Fengjiang Qin, Zhigang Zhang, Bo Xie, Rui Sun

**Affiliations:** Key Laboratory of New Technology for Construction of Cities in Mountain Area, Ministry of Education, School of Civil Engineering, Chongqing University, Chongqing 400045, China; qinfengjiang@cqu.edu.cn (F.Q.); zhangzg@cqu.edu.cn (Z.Z.); m13678426488@163.com (B.X.)

**Keywords:** engineered cementitious composite (ECC), flexural behavior, piezoelectric transducer, damage detection

## Abstract

The use of engineered cementitious composite (ECC) has attracted extensive attention in recent years because of the highly enhanced ductility owing to its unique strain-hardening behavior. In this paper, an electromechanical impedance-based technique is used to monitor the structural damage of RC beams strengthened with an ECC layer at the tensile zone. To achieve this purpose, three specimens are tested under bending loads to evaluate the proposed damage detection methodology. Five externally bonded PZT transducers are uniformly distributed at the surface of the ECC layer of the beams to measure the output conductance signatures in a healthy state and in different damage scenarios induced by different load levels. Test results showed that discrepancies exist between the signals measured in the intact state and each damage state, which can be used to evaluate the structural integrity changes. To assess the damage of ECC-concrete composite beams quantitatively, the statistical scalar index-root mean square deviation (RMSD) is used as the index, which can be calculated from the variations of conductance measurements of PZT sensors. The damage index values of the uniformly distributed PZT sensors provided cogent evidence of damage and revealed the evolution of structural damage. The crack patterns of beams at different damage levels are compared with the damage index values, and it shows the damage location can be derived from the measured conductance signatures of an array of PZT transducers.

## 1. Introduction

Concrete is widely used as a construction material because of its high strength and durability as well as the easy availability worldwide. Great attention has been paid in past decades to enhance the mechanical performance of concrete due to its inherent weaknesses, such as low tensile strength, brittle nature, and poor crack-resistance. In recent years, randomly-distributed short fibers with specific properties (usually polyvinyl alcohol (PVA) or polyethylene (PE)) have been added to the cement-based matrix to develop a new kind of high performance of polymer fiber reinforced concrete (called engineered cementitious composite (ECC)) [1,2,3,4,5]. Although only a low fiber volume (typically lower than 2%) is employed, the tensile strain capacity of material can be improved significantly due to the interactions between the composite fibers and the cement matrix. The tensile strain of ECC normally ranges between 3~8%, which is hundreds of times that of conventional concrete [6,7]. Unlike the brittle nature of concrete, the fiber bridging effect of ECC within cracks allows the crack width to be controlled below 100 μm, and thus prevents the appearance of millimeter-sized localized fracture cracks which are usually observed in normal concrete [8,9].

Numerous studies have been conducted to assess the mechanical behavior of steel reinforced ECC members (R-ECC). Superior ductility is observed because of the interfacial interactions between randomly-distributed fibers and cement matrix, which allow ECC to bear tensile, flexural and shear loads [10,11,12]. Hence, the brittle failure modes such as shear failure and bond-splitting could be well controlled by using ECC materials. The small-spacing micro-cracks of ECC enable it to resist the aggressive environment and especially the corrosion of steel reinforcement [13,14,15]. Besides, the unreacted cementitious particles at the location of cracks are likely to hydrate continually under moisture environments and thus produce new hydration products to fill the micro-cracks, that is a self-healing characteristic which can be used for rehabilitation of damaged structures [16,17,18]. Moreover, performance assessments of ECC members and structures subjected to monotonic, cyclic and seismic motions, can be performed experimentally [19,20,21], which demonstrates that the stiffness, strength, ductility and energy absorption of ECC structural members have a significant improvement than that of conventional RC structures. In a word, due to its excellent mechanical properties, ECC is a promising material to be applied in construction engineering [22,23,24] in order to enhance the structural performance against cracks, impact, fatigue loading and earthquake motions.

With the application of ECC materials in construction engineering, the damage detection of ECC structures has become an important issue to ensure their structural safety during their service life. By monitoring structural damages in real-time, the operational conditions of structures can be evaluated to prevent structural failures and reduce the risk of catastrophic accidents. In recent years, an impedance-based structural health monitoring (SHM) technique by using piezoelectric ceramic (PZT) sensors has attracted extensive attention due to its superior actuating/sensing properties, high sensitivity, economical cost and free model [25,26,27]. The PZT transducer, that serves as both actuator and sensor simultaneously, is usually internally embedded or bonded on the surface of a host structure. It has been theoretically proven that the electrical impedance of PZT sensor can be expressed in terms of the mechanical impedance of the host structure, which is directly related to the changes of structural integrity [28], thus, indicators extracted from impedance signatures can be used to assess structural damages. By applying PZT sensors, numerous experimental studies have been conducted to monitor damages of pipe systems [29], bolt joints [30], RC structures [31], bridges [32] and composite structures [33,34]. Numerical simulations are also carried out to study the sensitivity and accuracy of the impedance analysis and to identify structural damages according to the variation of impedance spectra [35,36,37].

Practical applications of the impedance-based methodology have demonstrated the feasibility of PZT sensor for SHM. However, to the authors’ best knowledge, few works were concentrated on the damage assessment of RC structures with ECC materials. Therefore, this study is focused on the damage evaluation of ECC-concrete composite beams subjected to bending loads. To this end, three RC beams were prepared for four-point bending load tests, and PZT transducers were externally bonded at the surface of the ECC layer using an epoxy adhesive. The electrical conductance spectra of each PZT sensor is measured under different structure damage scenarios. Thereafter, a statistical damage index RMSD, which is derived from the variations of measured signatures, is deployed to quantitatively assess the appearance and progression of structural damage as the applied load increases.

## 2. PZT Sensing Methodology for SHM

PZT sensors are made of piezoelectric material which is able to mutually transfer mechanical vibration and alternating current due to the direct and inverse piezoelectric effect. Actuating and sensing of PZT sensors would be simultaneously activated as long as an alternating voltage field is applied across the poling direction on the sensor. When a PZT transducer is internally embedded or externally bonded with a host structure, the mechanical vibration would interact with the PZT sensor and the self-sensing property makes it able to measure the output electrical impedance in a particular frequency range using an impedance analyzer (or LCR meter). The measured impedance signals are coupled to both the electrical impedance of the PZT sensor and the mechanical impedance of the host structure, in which the second item is directly related to structural integrity. Therefore, any damages in structures can be evaluated according to the variation of PZT output signatures. 

Considering the constitutive equation and polarization direction along the thickness of a PZT patch, Liang [28] developed the formulation of a one-dimensional model to represent the dynamic interaction between PZT transducer and structure using a simplified single-degree of freedom system. The electrical admittance signatures *Y(ω)* can be expressed as:

(1) $$Y(\omega )=G(\omega )+jB(\omega )=j\omega \frac{b_{PZT}l_{PZT}}{e_{PZT}}({\bar{\varepsilon }}_{33}^{T}-\frac{Z_{s}(\omega )}{Z_{s}(\omega )+Z_{a}(\omega )}d_{31}^{2}{\bar{Y}}_{11}^{E})$$

where *G(ω)* (real part) and *B(ω)* (imaginary part) represent the electrical conductance and susceptance, respectively. *ω* is the angular frequency of the excitation voltage. ${\bar{Y}}_{11}^{E}$ denotes the complex Young’s modulus in the length direction of a PZT patch with width, length and thickness designated by *b_PZT_*, *l_PZT_* and *e_PZT_*, respectively. ${\bar{\varepsilon }}_{33}^{T}$ and *d*_31_ are the electric permittivity in thickness direction and coupling piezoelectric constant which are defined by the constitutive relationship of piezoelectric materials. *Z_a_*(*ω*) and *Z_s_*(*ω*) are the mechanical impedance of the PZT sensor and host structure.

The electrical admittance of PZT transducer *Y(ω)*, which is composed of electrical conductance *G(ω)* and susceptance *B(ω)*, is in function of the mechanical impedance of the host structure as shown in Equation (1). Hence, any variations of admittance spectra directly reflected in the structural properties, such as stiffness, mass and damping, can be employed for damage detection. It is noted that the electrical conductance *G*(*ω*) (real part of admittance) can reflect the changes of structural integrity, while the susceptance *B*(*ω*) is more sensitive to the damage of PZT sensor itself. Therefore, in this work, electrical conductance signature is used for structural health monitoring due to its better indication of any structural property changes. 

The selection of the frequency range would influence the effectiveness of the proposed electromechanical admittance (EMA) methodology and the damage detection ability greatly depends on the successful frequency range selected rather than the voltage of excitation. Usually, a hundreds of kilohertz of frequency range is adopted due to the fact that the stress wavelength should be smaller than the size of the damage present in the host structure, which is one of the major concerns. From various studies of which the selected frequency ranges have been up to 1000 kHz [27], it is found that a lower frequency range results in a larger sensing area and a higher frequency range results in a smaller sensing area. However, it is also noticed that a higher frequency range is more sensitive to detect incipient and minor damages at an early stage. Hence, the frequency range should be defined by finding a balance between sensing area and ability of damage detection according to the requirements in each real case. Furthermore, the EMA signature with a frequency range higher than 600 kHz contains more information related to the physical properties of the PZT patch and the interfacial bond layer rather than host structure itself [38,39,40]. Therefore, in this work the conductance signature of a frequency band between 0–600 kHz is adopted for subsequent analysis and structural health monitoring of ECC-concrete composite beams.

## 3. Experimental Program

### 3.1. Material Properties

The main ingredients of ECC used in this work consisted of Portland cement (CEMI-52.5), fly ash, silica sand, PVA fibers, polycarboxylate-based high-range water reducing admixture (HRWRA), and water. The average size of silica sand is 150 μm with the maximum size of 200 μm. A total volume of 2% polyethylene (PE) fibers, that has a length of 12 mm and diameter of 26 μm, were added in the mortar paste for mixing until disperse well. The mixture proportions of ECC materials are listed in Table 1.

**Figure 1:**
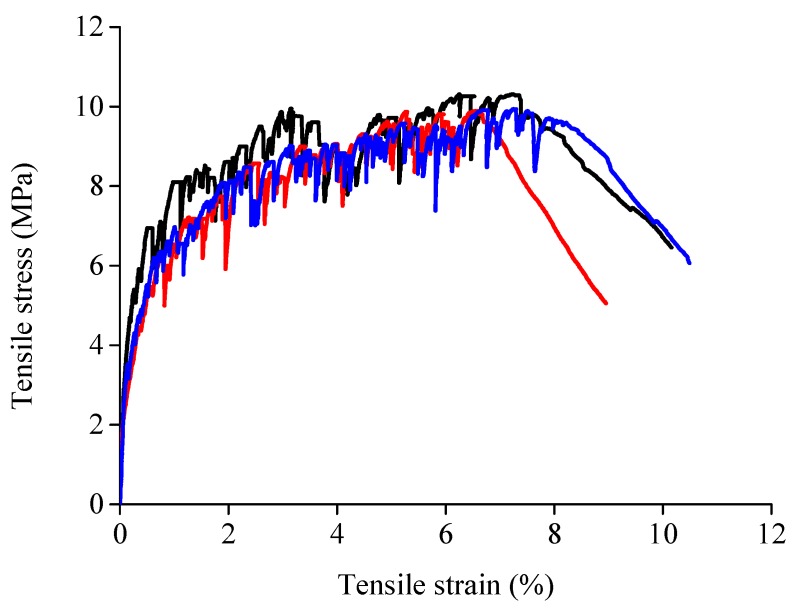
Measured tensile stress-stain curves of ECC.

To investigate the tensile stress-strain relationship of the ECC material in this study, three dogbone-shaped specimens were prepared and linear variable displacement transducers (LVDTs) were used to test the elongation of specimens within the gauge length. The detailed dimensions of the specimens and the test set-up can be found in [41,42]. The loading rate of the tensile test was controlled as 0.5 mm per minute, and the tensile strain was calculated according to the average value of the LVDTs. The measured stress-stain curves of ECC from uniaxial tensile tests are presented in Figure 1. As shown, all tensile stress-strain curves of ECC used in this study exhibit a very evident strain-hardening phenomenon and high tensile strain capacity. The average tensile strain of ECC reaches 7.27% with variance of 0.38%, which is nearly 700 times that of conventional concrete. On the other hand, due to the fiber bridging effect, the ultimate tensile strength of ECC is 9.92 MPa with variance of 0.31 MPa. This value is rather high as the tensile strength of concrete with compressive strength of C80 grade is normally below 3 MPa. It is noted that the average compressive strength of ECC used in this study is 79 MPa.

### 3.2. The Test Specimens

**Figure 2:**
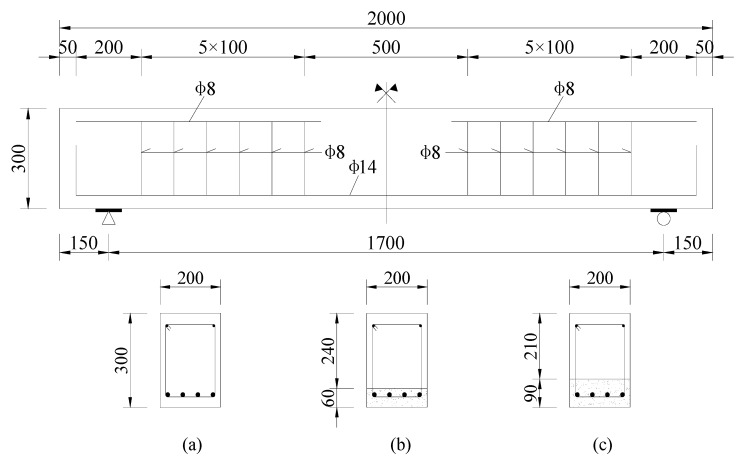
Longitudinal and cross section of the test beams: (**a**) RC beam, (**b**) BE1, (**c**) BE2 (unit: mm).

A total of three beams were tested in this work. Two of them, named BE1 and BE2, respectively, were strengthened with a 6 cm and 9 cm ECC layer at the bottom. One RC beam without an ECC layer was tested as the reference beam. A timber mold with the dimensions of 2000 mm × 300 mm × 200 mm was established to prepare the test specimens. All beams were cast to have the same cross section size of 300 × 200 mm, differing only in the thicknesses of the different ECC layers that were 6 cm and 9 cm, respectively, as shown in Figure 2. The reinforcement ratio of the three specimens was 1.18%, in which four longitudinal bars of 14 mm nominal diameter were distributed in the tension area as steel reinforcement.

### 3.3. Test Description

**Figure 3:**
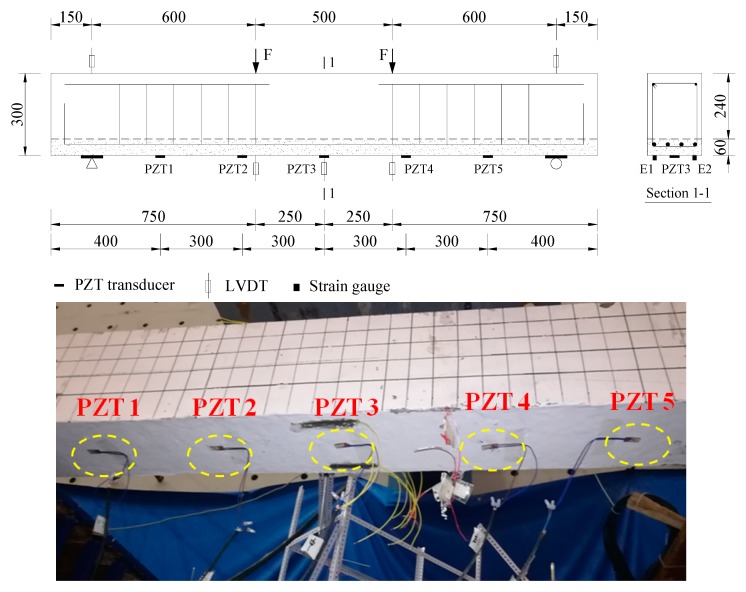
Schematic test setup and layout of LVDTs and PZT sensors on the beams (unit: mm).

The schematic test setup is shown in Figure 3. A servo-hydraulic testing machine was used to perform four-point bending tests through a spreader steel beam. The distance of the two loading points was 500 mm, and the length between loading point and end support was 600 mm. Three LVDTs were placed at the middle of beam to measure the deflection of the midspan and two loading points. Two LVDTs were installed to monitor the settlement of end supports which were placed at a distance of 150 mm from the ends of the beam. Five PZT sensors with dimensions of 10 mm × 10 mm × 1 mm were externally bonded on the bottom surface of each beam by using an epoxy resin adhesive. Two strain gauges, E1 and E2, were placed at the middle section of the test beams.

It is noted that the PZT sensors bonded at the bottom of RC beams are easily broken with the expansion of the crack width nearby. However, the micro-cracks of ECC reinforced concrete beams with low crack width would steadily progress in a small spacing of 3–6 mm due to the excellent crack control ability of ECC materials, which makes it able to maintain the integrity of PZT sensors bonded on ECC layers.

**Figure 4:**
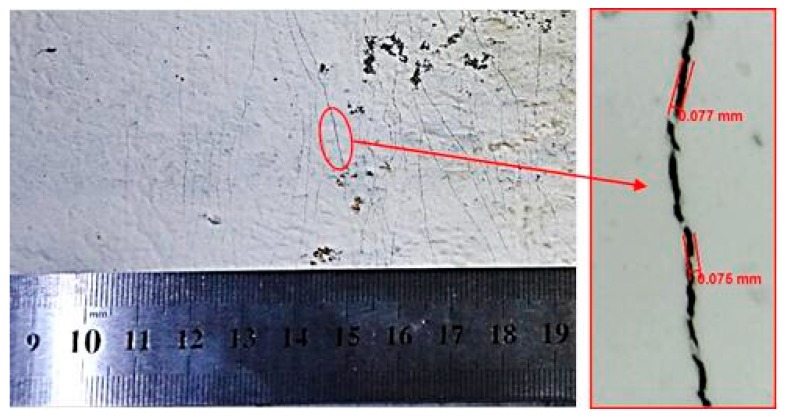
Maximum crack width on ECC layer during loading test.

Figure 4 displays the typical distribution of micro-cracks of ECC layer at the bottom of the RC beams in this study, and the maximum crack width at ultimate load is less than 0.08 mm. Therefore, in this study, the PZT patches were only bonded on the surface of ECC layers of two ECC-concrete composite beams BE1 and BE2, and the conductance signatures of these transducers were measured under several damage scenarios by using an LCR meter. The RC beam was only tested under bending loads and used as reference beam to compare the flexural behaviors of BE1 and BE2. Furthermore, according to previous studies [25,27], a single PZT transducer is more effective to detect structural damages within a sensing range of 400 mm in the electromechanical conductance based approach. Hence, five PZT patches were located at the surface of ECC layers with a distance of 300 mm between each sensor as shown in Figure 3. 

A preloading procedure was firstly conducted for the specimens. All of the test beams were subjected to bending loads with an increment of 3 kN up to 6 kN, and the bending loads were released to check the structural condition of specimens. Then, the applied loads on the specimens were gradually increased up to the ultimate bearing capacity of beams in order to generate different levels of structural damages. After that, the bending loads were released gradually by controlling the servo-hydraulic testing machine. During the loading process, the deflection at midspan of three test beamswere measured by LVDTs as shown in Figure 3, and they were continuously read and recorded by using a data acquisition system.

**Figure 5:**
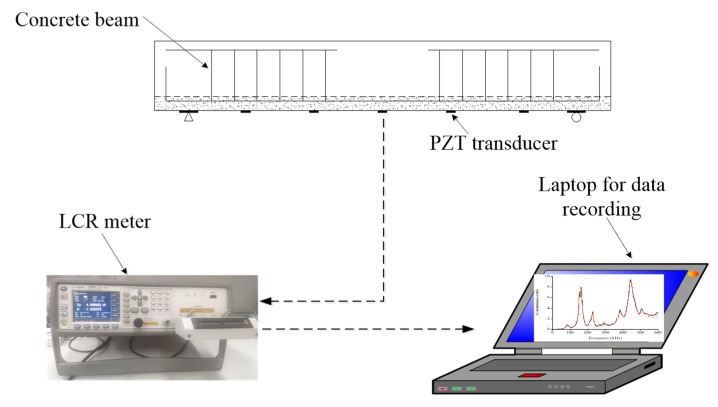
Schematic diagram of the experimental setup for electrical conductance acquisition.

Internal embedded PZT patches, also known as “smart aggregate”, were used to detect structural damages in previous studies [43,44,45,46], but they must be deployed prior to concrete casting and are not applicable for existing structures. Therefore, in this work PZT transducers were externally bonded on the bottom surface of beams in order to monitor the appearance and evolution of structural damages during its service time. As shown in Figure 5, an LCR meter (model E4980A, Agilent Technologies, city, state abbrev if USA, country) was employed to measure the conductance signatures of five uniformlydistributed PZT patches of each test specimen under different damage scenarios. To achieve the purpose, a sinusoidal voltage signal with 1 volt amplitude was generated by the LCR meter to apply on the surface of PZT patches to sweep over a defined frequency range. The output electrical conductance of PZT sensors were measured to detect the damages induced by changes in the mechanical impedance of host structure as shown in Equation (1). The alternations in the output signals become indicative of the structural damages when the host structure is monitored by extracting the electrical conductance signals of PZT patches. Therefore, not only the appearance of damages in ECC-concrete composite beams can be detected but also the propagation can be assessed according to the measured electrical conductance signatures during the loading test.

## 4. Test Results and Discussion

### 4.1. Flexural Behavior

**Figure 6:**
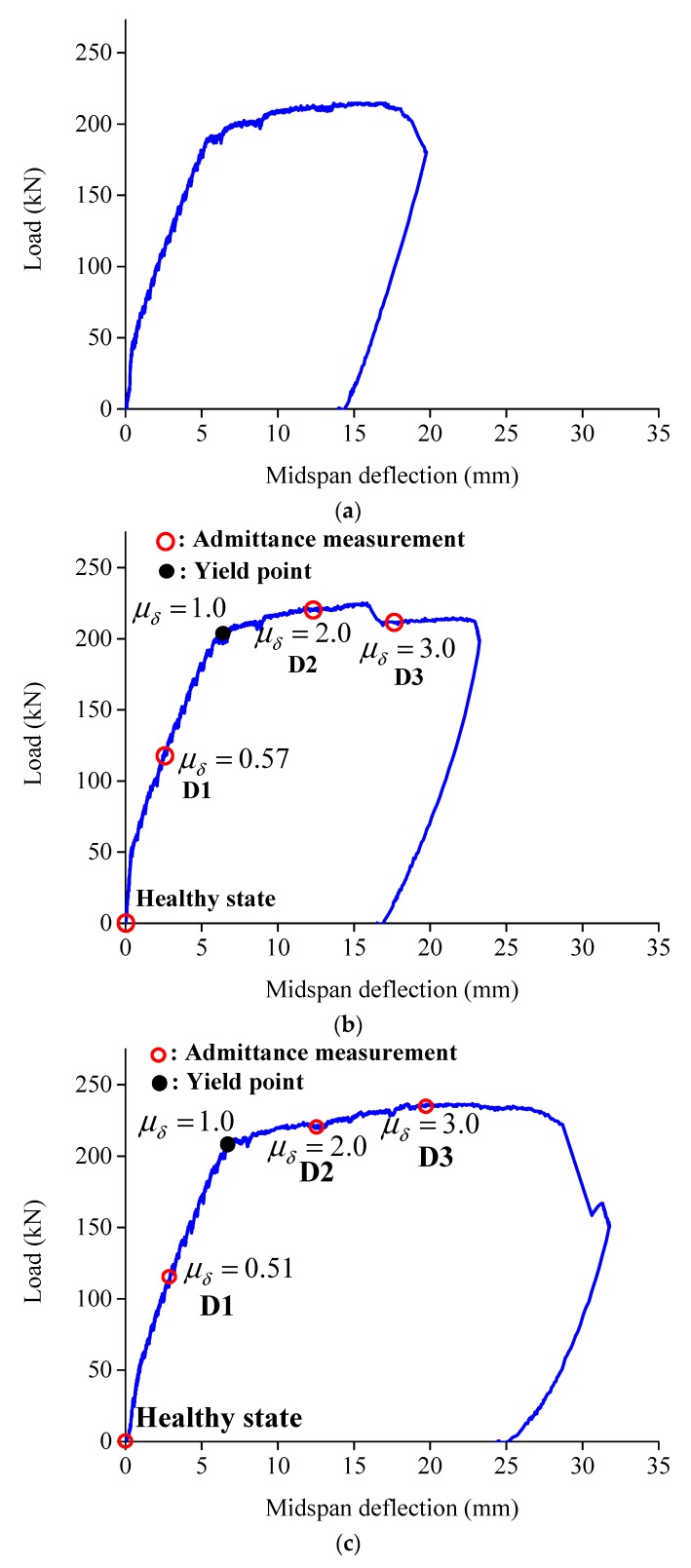
Loadmidspan deflection of the three test specimens: (**a**) RC beam; (**b**) BE1; (**c**) BE2.

As shown in Figure 6a–c, the relationships of load and midspan displacement of three test specimens are obtained by using LVDTs installed at the center of the beams. The first cracks of the RC beam, BE1 and BE2 appeared at loads of 45 kN, 51 kN and 56 kN, respectively. The micro-cracks of BE1 and BE2 perpendicular to the direction of maximum principal stress were spaced tightly along the ECC layer of the beams and crack width were smaller than that of the reference RC beam. Then, the tensile longitudinal steel of BE1 and BE2 yielded at 205 kN and 210 kN with related midspan deflection of 6.2 mm and 6.7 mm, respectively, while the yielding load of RC beam was 191 kN with a midspan deflection of 5.5 mm. Afterwards, the midspan deflection increased quickly while the applied load increased slowly until the final failure of the three test specimens, and the number of cracks of BE1 and BE2 increased with the increase in the applied load while the crack width nearly performed to be constant, owing to the excellent capability to resist crack growth of ECC materials. 

By contrast, the crack spaces and crack width of RC beam are larger than those with ECC layers at bottom, and the crack width would increase as the cracks propagated towards the compression zone during the loading test. According to the test results, there is no obvious improvement of load-carrying capacity of RC beams by using an ECC layer as strengthening. Nevertheless, the midspan deflection of BE1 and BE2 related to ultimate load are 23.1 mm and 28.8 mm, respectively, which represent a significant increase of 26.2% and 57.4% compared with 18.3 mm of the reference RC beam. This is mainly attributed to the fiber bridging and excellent crack control ability of the PVA-ECC matrix in the ECC layer according to its strain-hardening response. The load and midspan deflection correspond to yielding and ultimate points are summarized in Table 2.

**Figure 7:**
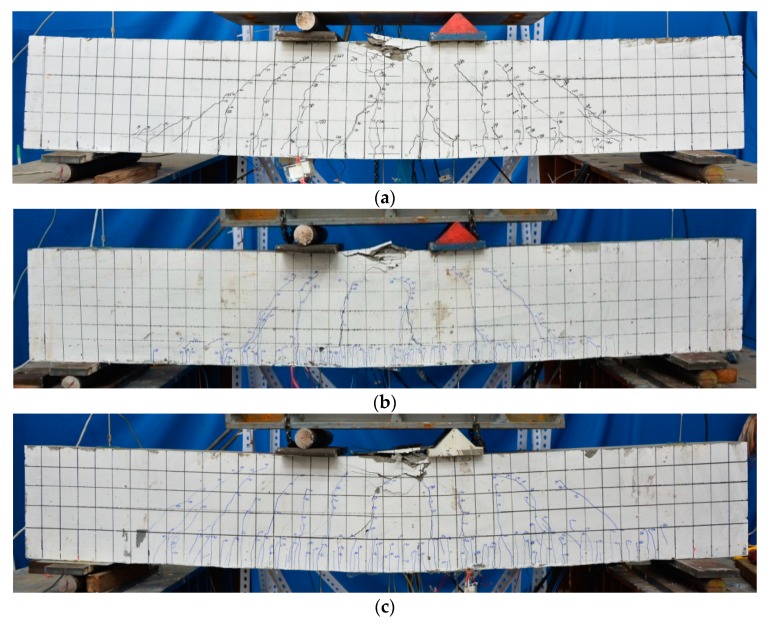
Failure modes of three test specimens: (**a**) RC beam; (**b**) BE1; (**c**) BE2.

Failure modes of the test specimens under bending loads are displayed in Figure 7a–c. Similar failure modes of three specimens were observed that the concrete in compressive zone crushed at the ultimate load after the yielding of steel reinforcement resulting in the final failure of beams.

Furthermore, it is clear that multiple micro-cracks were distributed in a tightly space in the ECC layer at the bottom of BE1 and BE2 as shown in Figure 7b,c, which is distinctively different with those of the conventional RC beam.

**Figure 8:**
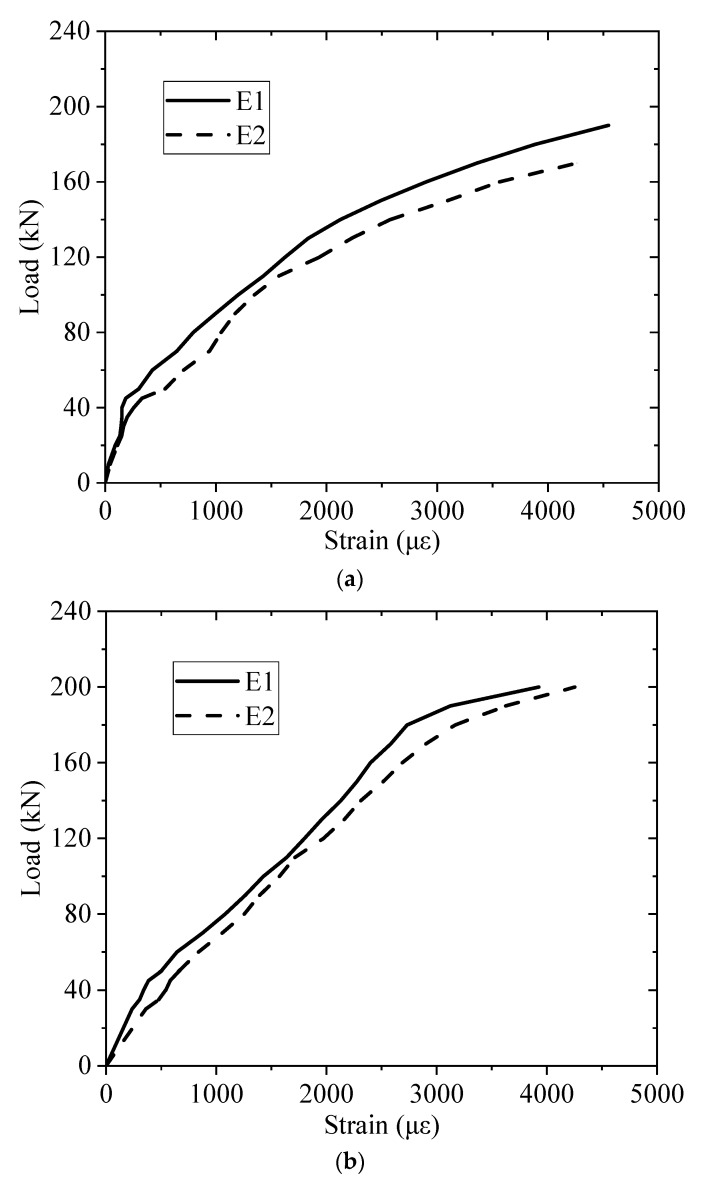
Load-strain relationship of ECC layer at the midspan of beams: (**a**) BE1, (**b**) BE2.

Owning to the micro-cracks and small crack width of the ECC layer, the two strain gauges (E1 and E2) installed at the middle section of BE1 and BE2 are able to be used to measure the ECC strain at midspan as shown in Figure 3. The load-strain relationships of the ECC layers of BE1 and BE2 are displayed in Figure 8. The ECC strain at the bottom of two beams increases as the bending loads increase until the final failure of the test beams. The average ultimate ECC strains of BE1 and BE2 are 4400 and 4100 με respectively, which demonstrates the excellent ductility of ECC materials.

### 4.2. Measured Conductance Signatures

To measure the conductance spectra of the five PZT patches of BE1 and BE2, four different damage levels of ECC reinforced concrete beams aredefined in Table 3 and described as follows:

(1) The intact beam before applying bending loads is defined as “healthy state (D0)”;
(2) At midspan deflection *δ* = 3 mm before the yielding point of steel reinforcement, which relate to a deflection ductility *μ_δ_* = 0.48 and *μ_δ_* = 0.45 for BE1 and BE2, respectively (*μ_δ_* = *δ*/*δ_y_*, the midspan deflections related to yielding point *δ_y_* are 6.2 mm and 6.7 mm for BE1 and BE2 as shown in Table 2). This state is defined as “damage level 1 (D1)”.
(3) At midspan deflection δ=12.4 and 13.4 mm after steel yielding for BE1 and BE2, that corresponded to *μ_δ_* = 2.0 are defined as “damage level 2 (D2)”, and at the same way, *δ* = 18.6 and 20.1 mm corresponded to *μ_δ_* = 3.0 for BE1 and BE2, respectively, are defined as “damage level 3 (D3)”.

**Figure 9:**
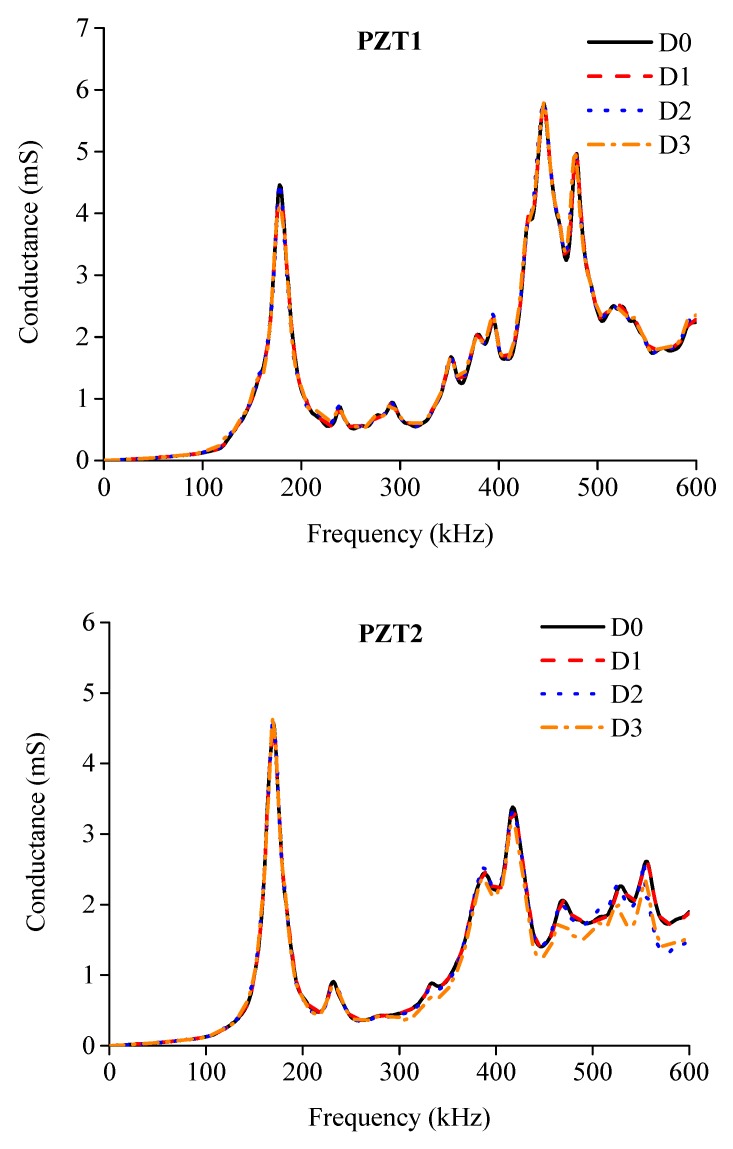

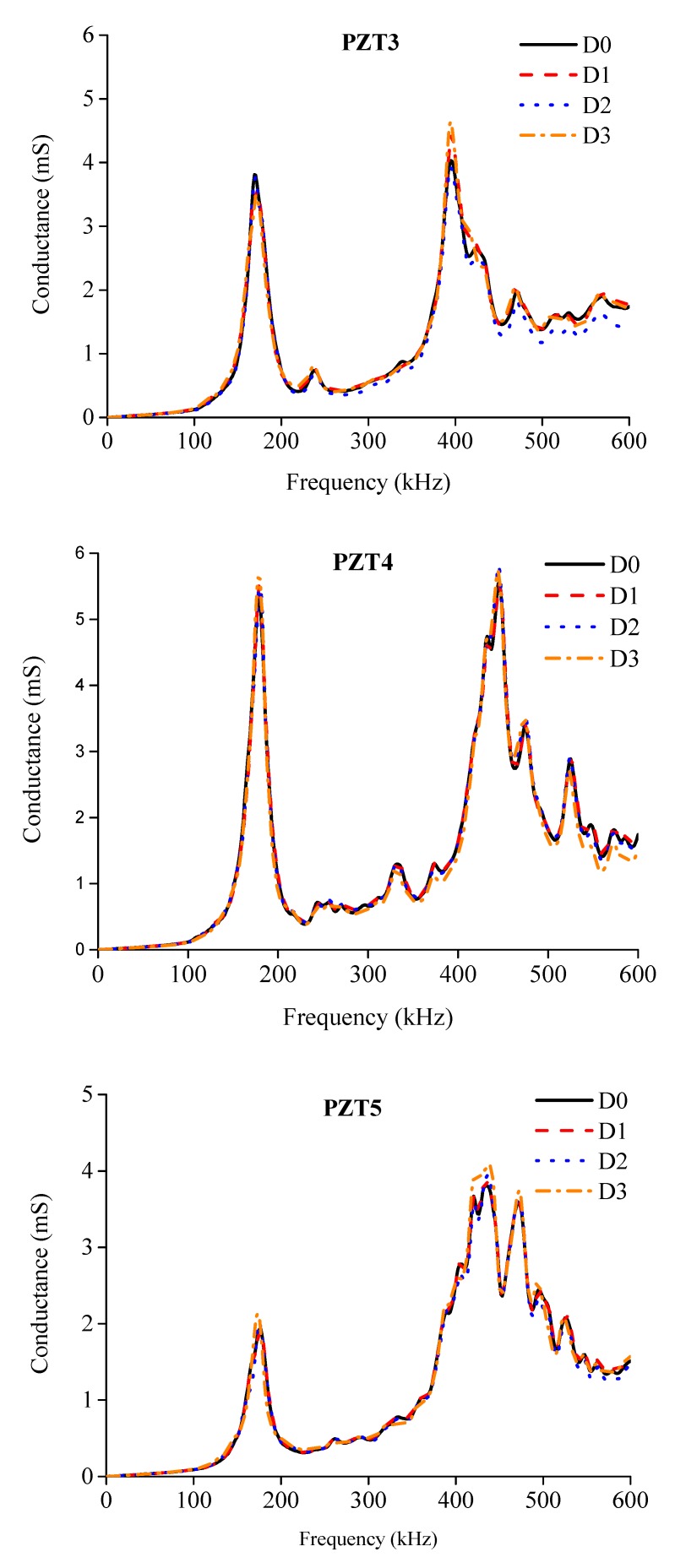
Comparisons of electrical conductance signature of each PZT sensor under different damage levels of BE1.

**Figure 10:**
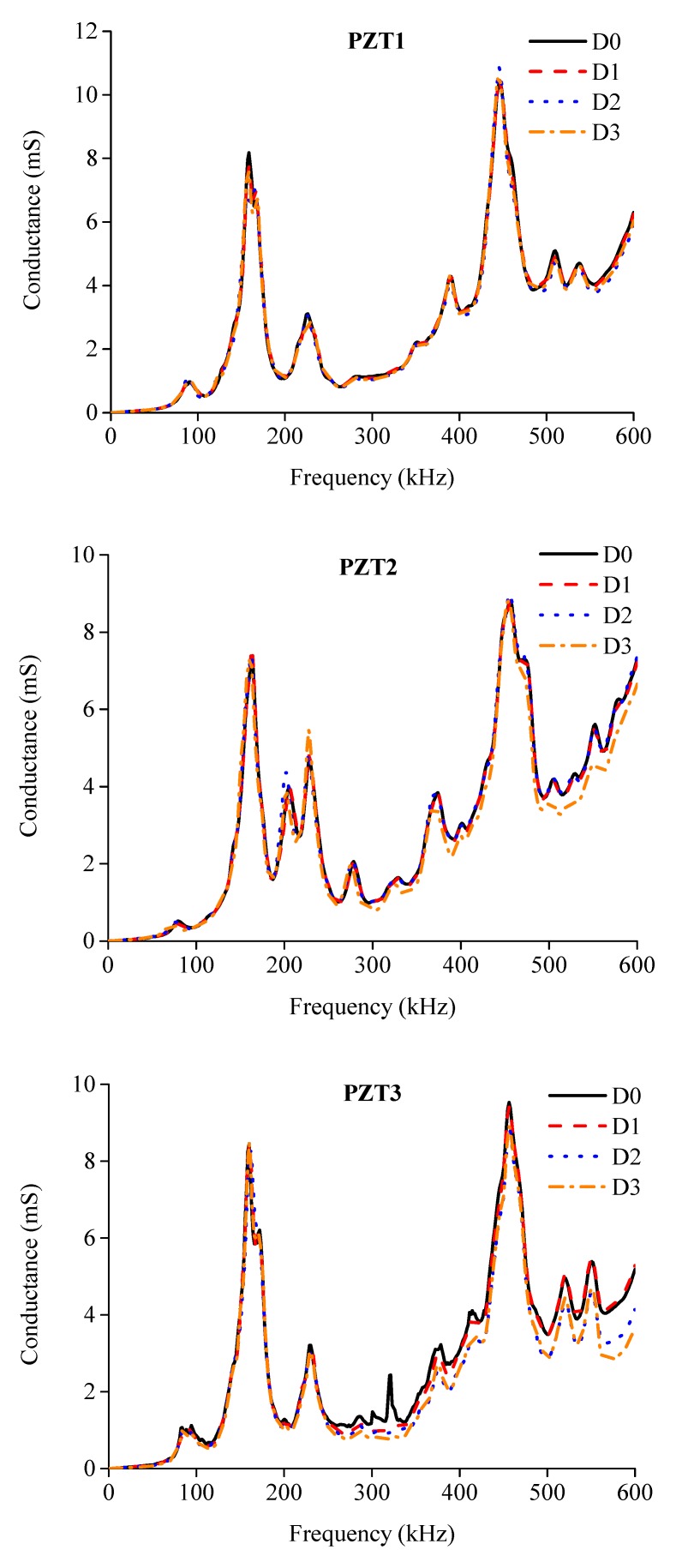

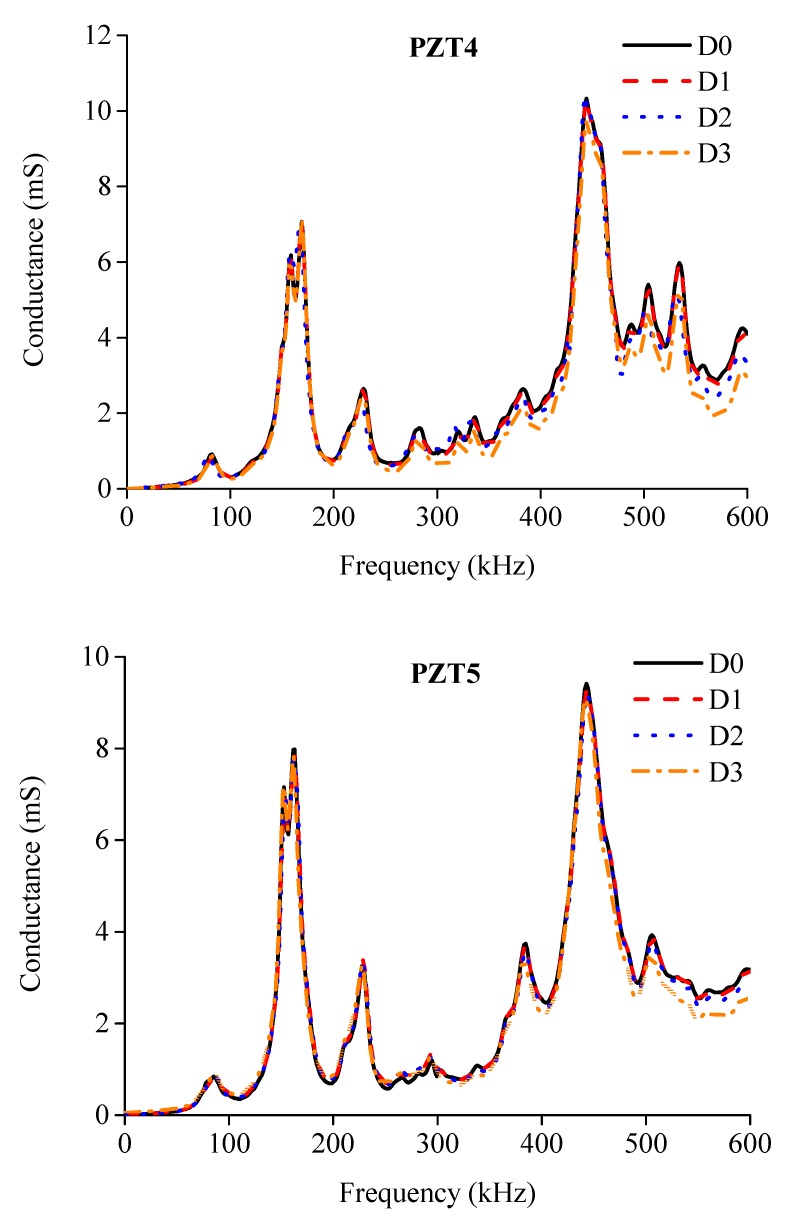
Comparisons of electrical conductance signature of each PZT sensor under different damage levels of BE2.

Electrical conductance spectra of the externally bonded PZT patches were first measured at the beginning of the test for BE1 and BE2, which were under healthy state D0. After that, when the three damage levels D1, D2 and D3 were achieved with the increase of applied load as illustrated in Figure 6b,c during the test, the conductance signatures were also captured by using the LCR meter. Figure 9 and Figure 10 show the conductance signatures measured by the PZT transducers of BE1 and BE2 under different damage scenarios, from D0 to D3. The main resonant peaks around 100–200 kHz and 400–500 kHz can be explained by the electromechanical coupling effects. 

It should be noted that variations of temperature and humidity would affect the measured conductance signatures of PZT patches and result in erroneous detection results regarding the structural integrity. Considering the experimental tests in this work were conducted at almost constant temperature and humidity, thus, the influences of environment were neglected in the proposed work. 

Discrepancies among different damage scenarios can be observed which reflect the variations of structural integrity under different loading levels. It can be observed that the variations of conductance spectra at a higher frequency range are greater than those in lower frequency range. This is mainly due to the high sensitivity of conductance spectra in high frequency band to detect structural changes, which have been confirmed in previous studies [29]. Furthermore, it is also found that by only observing the variations in conductance signatures it is difficult to conduct damage assessment to different damage levels.

According to the EMA technique adopted in this work, the measured conductance before damage (initial state D0) and after damage (state D1, D2 and D3) will change as the applied load increases. Root mean square deviation (RMSD), which is directly proportional to the severity of structural damages with a maximum value of 1 indicating severe damage, is typically used to obtain a quantitative evaluation of structural damages. The greater the damage, the greater the variation in the conductance spectra and, based on its formulation in Equation (2), the greater the RMSD value. By introducing the statistical scalar index, quantification for conductance variations induced by damage can be identified. Thus, RMSD index is deployed to assess the evolution of damage severity of ECC-concrete composite beams in this work:

(2) $$\mathrm{RMSD}(\%)=\sqrt{\frac{\sum_{1}^{N}{\left(Z_{k}-Z_{0}\right)}^{2}}{\sum_{1}^{N}{\left(Z_{0}\right)}^{2}}}\times 100,k=1,2,3$$

where Z_0_ represents the conductance of the PZT transducer at healthy state (D0), which is used as baseline signature. Z*_k_* is the measurement at different damage states, where the subscript *k* = 1,2,3 is related to the three damage levels D1, D2 and D3 defined in Table 3. *N* is the sampling frequency points of measurements.

**Figure 11:**
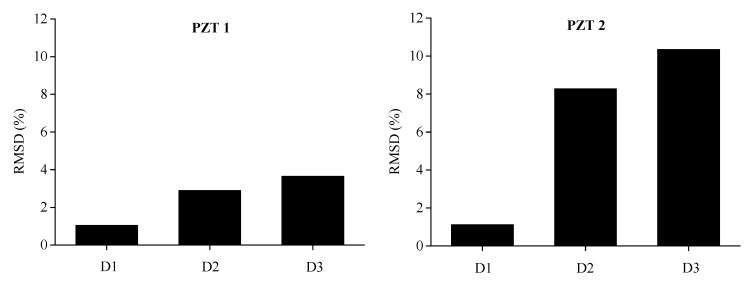

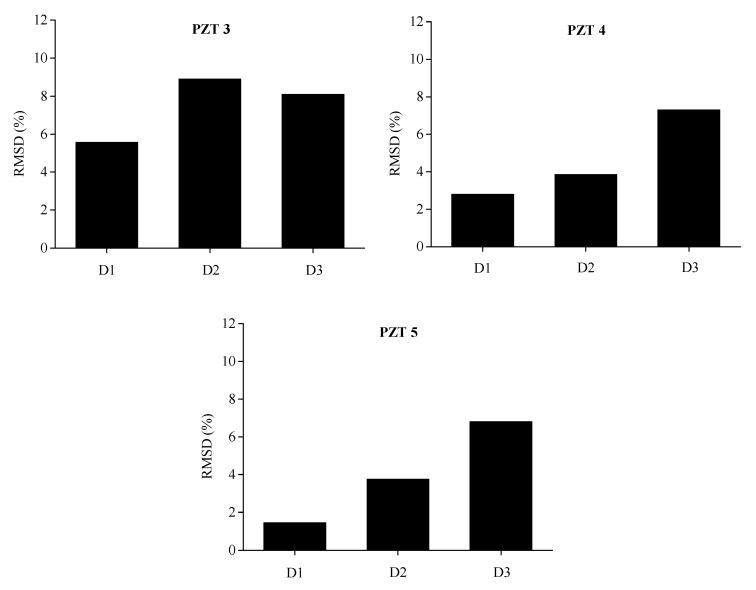
Experimental RMSD values of each PZT sensor for BE1 under different damage levels.

**Figure 12:**
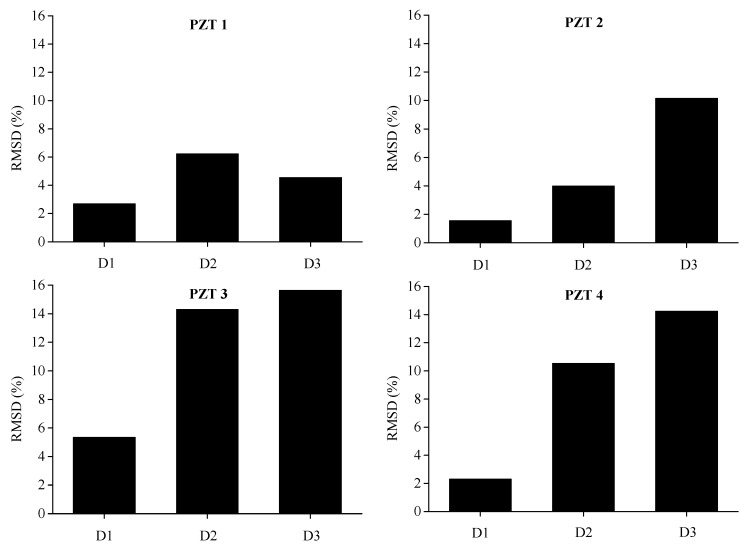

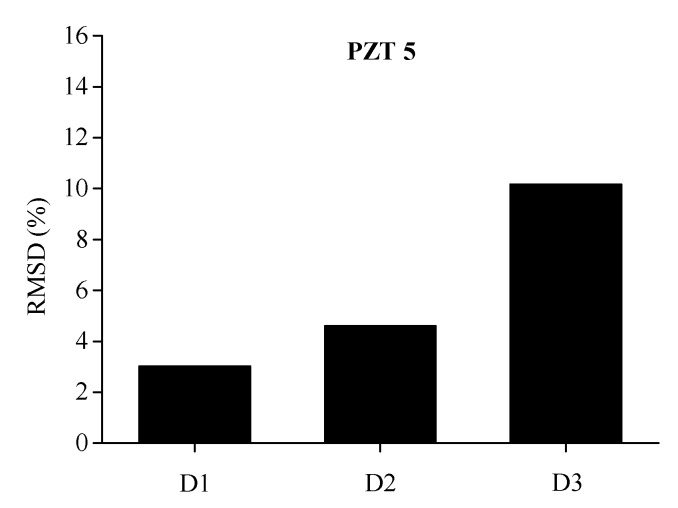
Experimental RMSD values of each PZT sensor for BE2 under different damage levels.

To comprehensively assess the structural damage of BE1 and BE2 monitored by PZT sensors that are distributed along the length of beam, RMSD value of each five sensors by using electrical conductance measured with a sweeping frequency range between 0 and 600 kHz are displayed in Figure 11 and Figure 12. It is clear that, more or less, with the increase of damage level, the RMSD values evaluated from the conductance measurements in most of the PZT transducers are ascending from D1 to D3.The induced structural damages, such as concrete cracking and steel yielding, would enlarge with the increase of bending loads during the experimental test. Therefore, the progressive growth of damage levels is confirmed by the increase of damage indicators calculated from conductance measurements, which corresponds quite well with the real damage case during the bending test. Furthermore, it is noted that slight decreases are observed for PZT 3 of BE1 from D2 to D3 and PZT 1 of BE2 from D2 to D3, this might be attributed to the disturbance during the test, e.g., measurement noise and temperature variation. In general, the damage indicator predicts a reasonable quantitative trend that is compatible with the progressive growth of structural damages. 

**Figure 13:**
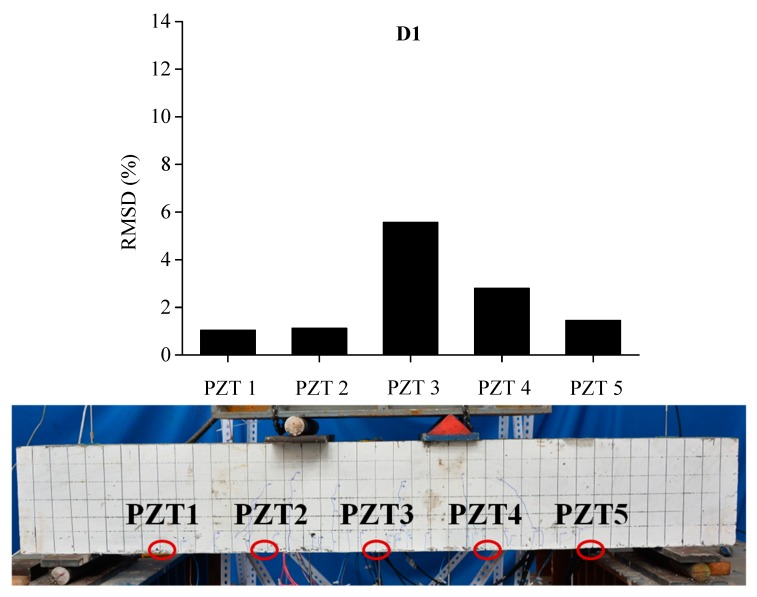
RMSD of each PZT sensor at D1 and the related cracking pattern of BE1.

**Figure 14:**
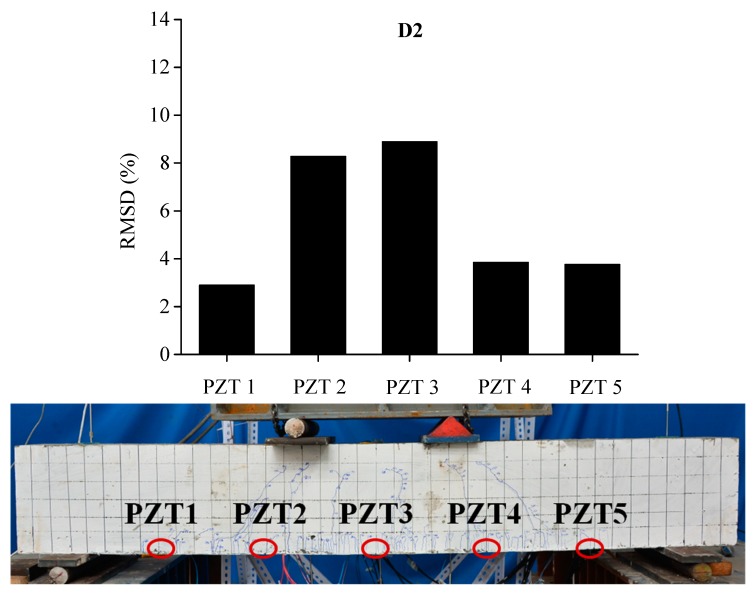
RMSD of each PZT sensor at D2 and the related cracking pattern of BE1.

**Figure 15:**
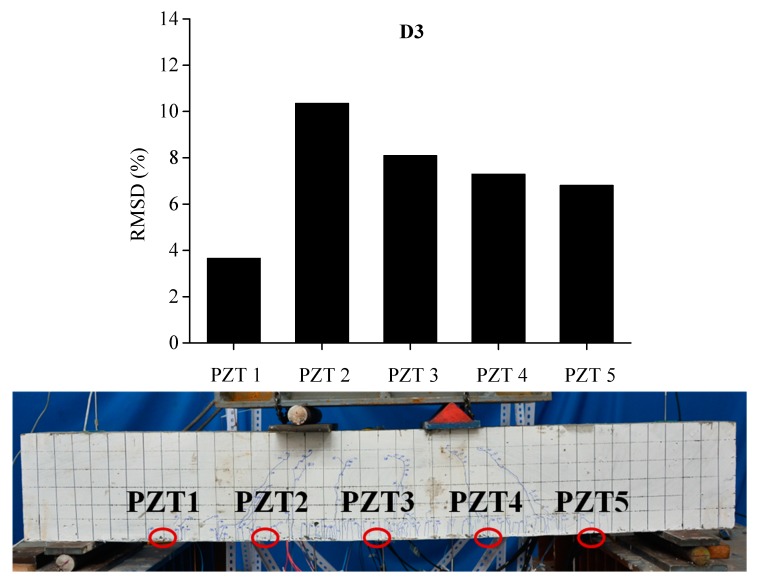
RMSD of each PZT sensor at D3 and the related cracking pattern of BE1.

**Figure 16:**
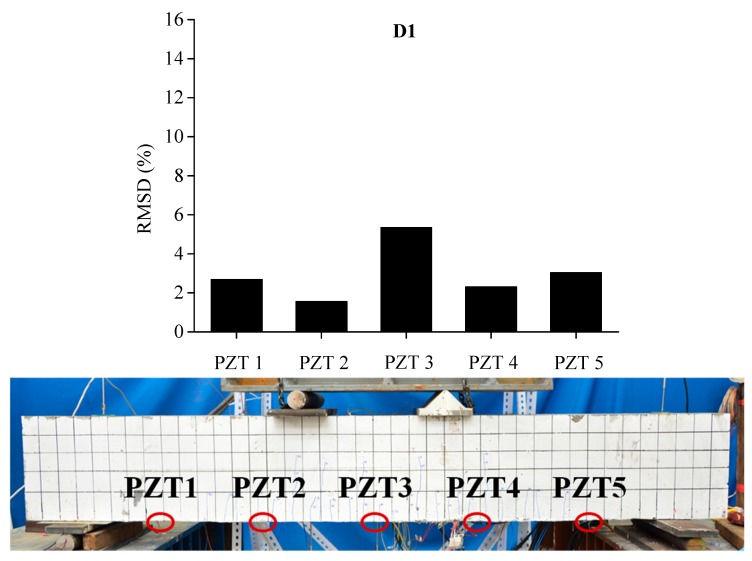
RMSD of each PZT sensor at D1 and the related cracking pattern of BE2.

**Figure 17:**
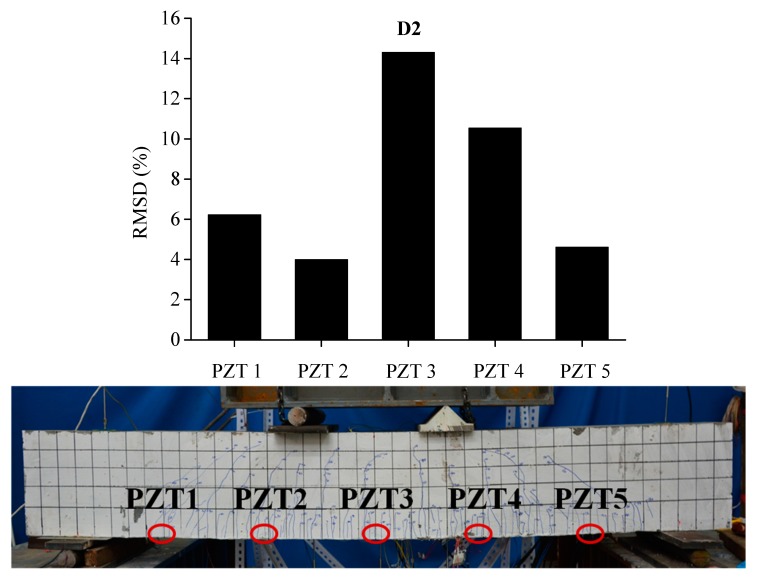
RMSD of each PZT sensor at D2 and the related cracking pattern of BE2.

**Figure 18:**
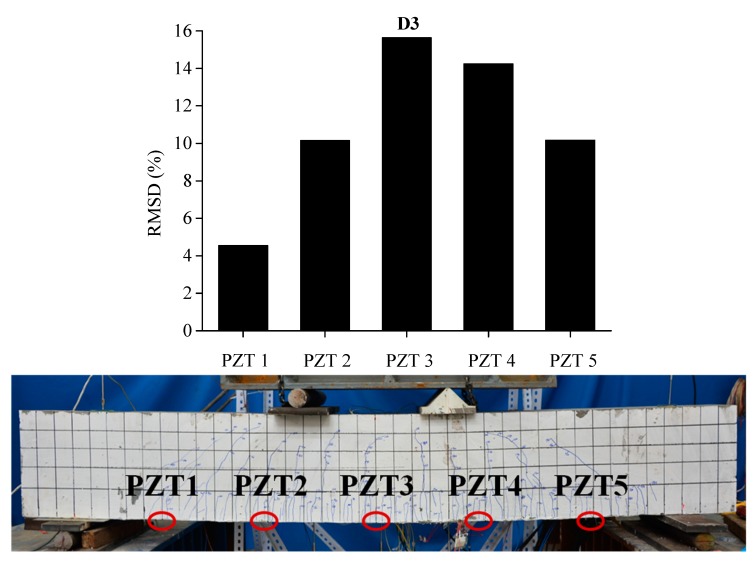
RMSD of each PZT sensor at D3 and the related cracking pattern of BE2.

In order to evaluate the influence of the location and severity of damage on conductance signatures and the ability of local sensing of PZT transducers, the RSMD values calculated from experimental measurements of each five PZT sensor uniformly located along the length of BE1 and BE2 are compared with the real crack patterns under different damage levels, as shown in Figure 13, Figure 14, Figure 15, Figure 16, Figure 17 and Figure 18. 

In Figure 13, the RMSD show low values for all sensors, which represents that BE1 has a low damage severity (D1). It can be noted that the damage index values of PZT 3 and PZT 4 at the midspan of beam are higher than those of PZT 1, PZT 2 and PZT5, which indicates the presence of crack damages at the midspan as cracking load is achieved. Afterwards, a significant increase of RSMD values can be observed for each sensor as the applied load increases as illustrated in Figure 14 and Figure 15, especially for PZT 2, PZT 3 and PZT 4 which are located in the vicinity of the midspan of BE1. This phenomenon can be explained by the increasing number of multiple micro-cracks in the ECC layers and the propagation of concrete cracks, and a high RMSD value of the PZT sensor indicates a high damage severity of the host structure and vice versa. General speaking, by comparing the damage index RMSD values of the distributed PZT sensors on the beam, the growth of damage severity would cause a relative increase of the RMSD value of the transducer nearest to the damage.

Similar phenomena are shown in Figure 16, Figure 17 and Figure 18 for the damage detection of BE2. PZT 3, that is located at the middle of beam, displays a higher RMSD value than the other sensors at damage level D1after initial cracking. Then, the expansion of induced damages caused by the applied load would influence the output measurements of PZT sensors and result in higher values of RMSD metric. In the same way, the RMSD values of PZT patches which are externally bonded in the neighborhood of middle section of BE2, such as PZT 2, PZT 3 and PZT 4, are higher than PZT 1 and PZT 5, which are placed near the two supports. 

According to the comparison between damage prediction and cracking patterns in a real case, it can be concluded that for an array of PZT transducers in ECC-concrete composite beams the structural damage alters the conductance measurements of a particular PZT sensor in inverse proportion to the distance from the damage location, which implies the nearest PZT is most influenced and the farther ones less. Taking into account the large scale of the problem and the uncertainties during the tests, RMSD value obtained from the variations of measured conductance signatures using an array of externally bonded PZT sensors seem to provide satisfactory reliability for the identification of damage severity at different damage levels.

## 5. Conclusions

An impedance-based approach was used to monitor the evolution of structural damage of ECC-concrete composite beams subjected to bending loads. To achieve this goal, three specimens were prepared for four-point loading tests, and their flexural behaviors were recorded using a data acquisition system. Meanwhile, three damage scenarios were selected to represent the different damage severities of the specimens during the test, and electrical conductance signatures in the range of 0–600 kHz were measured under different damage levels by using LCR meter. RMSD index is successfully used to conduct quantitative damage assessment for ECC-concrete composite beams. Following conclusions can be drawn from the experimental test:

(1) The ECC layer in the RC beams significantly increases the ultimate deflection due to its unique high ductility, which is beneficial to the structural safety.
(2) The maximum crack width is less than 0.08 mm and a small crack spacing of 3-6 mm is observed in this work, which demonstrates that the development of cracks in ECC-concrete composite beams is well controlled due to the fiber bridging effect of ECC materials. This is very helpful to prevent corrosion of steel reinforcements, in particular for aggressive environments.
(3) Damage detection has been successfully implemented using the statistical scalar index RMSD, which is calculated from the variations of PZT conductance measurements. The upwards trending of RMSD value confirms the increase of damage severity in the host concrete beam.
(4) The location of the occurred damages are also estimated by using an array of PZT sensors externally bonded on the ECC layer, since the RMSD value of PZT transducer placed near the region of higher damage severity is higher than those for other PZT sensors.

## Figures and Tables

**Table 1:** Mixture Proportion of ECC Mixture (by weight).

Cement	Fly Ash	Silica Sand	Water	HRWRA	PE Fiber
1	1	0.6	0.5	0.004	2% by volume

**Table 2:** Yield and ultimate load, and related midspan deflection of test beams.

Beam Type	Yielding		Ultimate		Failure Mode
	Load (kN)	Midspan Deflection (mm)	Load (kN)	Midspan Deflection (mm)	
RC beam	191	5.5	211	18.3	Concrete crushing
BE1	205	6.2	212	23.1	Concrete crushing
BE2	210	6.7	224	28.8	Concrete crushing

**Table 3:** Three different damage levels of BE1 and BE2.

Damage Level	*μ_δ_*	Damage Description
D0	0	Healthy state
D1	0.48(BE1), 0.45(BE2)	Flexural Cracking, Multiple micro-cracks in ECC layer
D2	2.0	Steel yielding, Multiple micro-cracks in ECC layer
D3	3.0	Steel yielding, Multiple micro-cracks in ECC layer
